# Treatment of Severe Atrophy with Juxta-Osseous Implants: A Systematic Review and Case Report

**DOI:** 10.3390/bioengineering13040386

**Published:** 2026-03-27

**Authors:** Alberto Gasbarri, Filippo Giovannetti, Giulia Caporro, Maurizio D’Amario, Renato Sperati, Ali Jahjah, Ettore Lupi, Mario Capogreco

**Affiliations:** 1 Department of Life, Health and Environmental Sciences, University of L’Aquila, 67100 L’Aquila, Italy; 2 Department of Maxillo-Facial Surgery, San Salvatore Hospital, ASL1 Abruzzo, Coppito, 67100 L’Aquila, Italy

**Keywords:** jaw atrophy, juxta-osseous implants, digital workflow, oral surgery

## Abstract

**Background**: Severe jaw atrophy limits traditional endosseous implantation, often necessitating complex regenerative procedures. Advances in digital planning and 3D printing have reintroduced custom-made subperiosteal (juxta-osseous) implants as a viable alternative. This study evaluates the clinical reliability and advantages of next-generation juxta-osseous implants. **Materials and Methods**: A systematic review was conducted in accordance with PRISMA guidelines across the PubMed, Scopus, and Web of Science databases. The search focused on English-language studies reporting on custom-made titanium juxta-osseous implants in patients with severe maxillary or mandibular atrophy. Methodological quality was assessed using the Joanna Briggs Institute (JBI) Critical Appraisal Checklist. Additionally, a representative clinical case of a 60-year-old female treated via a fully digital workflow is presented to illustrate the protocol. **Results**: Twenty-six articles were included, accounting for 147 clinical cases. Most patients exhibited Cawood and Howell Class V–VI atrophy. All identified treatments utilized integrated digital workflows, including CBCT imaging, CAD/CAM design, and additive manufacturing (SLM/DMLS) of medical-grade titanium alloy. Reported success rates exceeded 90%, with high primary stability enabling immediate or early loading protocols and high patient satisfaction. Complications were primarily limited to manageable soft-tissue dehiscence. **Conclusions**: Modern juxta-osseous implants represent a promising, minimally invasive alternative to bone grafting for severe atrophy, enabling rapid functional restoration in the short-to-medium-term. However, because current evidence is limited to clinical studies, these findings should be interpreted with caution. Long-term prospective trials are essential to establish definitive clinical predictability and standardized protocols.

## 1. Introduction

Severe bone atrophy remains a significant challenge in dental implantology. Progressive bone loss following tooth extraction—often resulting from periodontal disease, trauma, or congenital defects—compromises both the quantity and quality of bone required for the predictable placement of endosseous implants and long-term peri-implant health [1].

While conventional implants provide reliable outcomes for many partially or totally edentulous patients, their success depends on the adequacy of hard and soft tissues to ensure primary stability, osseointegration, and peri-implant health. In cases of severe bone atrophy, achieving implant-prosthetic rehabilitation can be difficult without resorting to extensive regenerative procedures, such as block bone grafts (inlay or onlay), guided bone regeneration (GBR), split-crest techniques, distraction osteogenesis, or sinus floor elevation [2]. Although these techniques, alongside other surgical interventions such as pterygoid and zygomatic implants, can restore function and aesthetics, they present notable limitations: they often require multiple surgical phases, prolonged healing periods, and high treatment costs, and they may not allow for immediate prosthetic rehabilitation. Furthermore, they are associated with higher complication and morbidity rates and require advanced surgical skills, and a lack of osseointegration can lead to total implant or prosthetic failure [3]. Consequently, alternative approaches—including pterygomaxillary and zygomatic implants—have gained prominence as viable solutions for severe atrophy, reducing the need for complex regenerative surgeries [4,5].

Modern innovations, such as advanced imaging systems, additive manufacturing, and the ability to create patient-specific customized implants, have significantly expanded the modalities available to clinicians for managing severe bone atrophy in cases where bone thickness precluded traditional endosseous implants. These innovations now enable the rehabilitation of patients with severe atrophy who cannot receive conventional endosseous treatments through the use of subperiosteal (juxta-osseous) implants [6,7].

The surgical approach to subperiosteal implantation has significantly evolved since its inception. The first clinical application was documented by Dahl in 1938, who proposed a framework resting on the bone surface [8]. This technique was further developed and refined during the 1950s and 1960s by pioneers such as Marziani and Linkow, whose contributions established the early clinical protocols for framework stability and mucosal support (as cited in Goh et al.) [9,10]. While these early attempts provided a fundamental basis for treating severe atrophy, they often faced challenges related to manual casting precision and long-term biological integration, which modern digital workflows now aim to overcome. Beyond purely technological evolution, the success of modern juxta-osseous systems is deeply rooted in their unique biological and biomechanical foundations. From a biological perspective, unlike endosseous implants that rely on intra-medullary osseointegration, subperiosteal frameworks interact primarily with the external cortical surface and the overlying periosteum. The use of medical-grade titanium promotes a stable interface with the basal bone, minimizing the chronic inflammatory responses that often plagued earlier non-biocompatible alloys [11,12,13,14].

From a biomechanical standpoint, the transition to digital workflows allows for the design of frameworks that strategically distribute masticatory loads across residual anatomical pillars, such as the canine buttress and the zygomatic region. This approach significantly reduces localized stress peaks on the atrophic cortex by utilizing rigid fixation through osteosynthesis screws placed in areas of higher bone density. Such computerized load management not only provides the primary stability required for immediate loading protocols but also helps prevent the peri-implant bone resorption typically observed in previous generations.

Thanks to progress in design and surgical protocols, this method currently represents an effective solution for prosthetic rehabilitation [8].

Although subperiosteal implants were already considered a treatment of choice for major atrophy at that time, success was hindered by an extremely invasive protocol. Surgery necessarily involved two phases: an initial massive bone exposure for manual impressions and a second phase for the insertion of the cobalt–chrome implant [12]. These implants were fabricated using rudimentary impressions—prone to the inaccuracies of impression materials—and conventional radiographs, which often resulted in designs lacking the precision required to accurately match the patient’s bone anatomy. This limited accuracy, combined with the non-integrable nature of the alloys used, led to an incongruous fit responsible for high rates of instability and complications, such as bacterial infiltration and dehiscence [12,13,14].

In contrast, modern subperiosteal implants benefit from advanced 3D imaging, digital planning, and CAD/CAM manufacturing. These technologies allow clinicians to produce highly accurate, patient-specific implants guided by case-specific anatomy that conform precisely to the patient’s bone contours. Furthermore, the digital workflow enables prosthetically driven planning, ensuring the final implant design supports the ideal rehabilitative outcome from the outset [15,16]. Additional advantages are provided today by the use of Selective Laser Melting (SLM) or Direct Metal Laser Sintering (DMLS)—additive manufacturing technologies (industrial 3D printing) that utilize high-power lasers to fuse metal powders layer by layer. Specifically, subperiosteal implants are produced in medical-grade V titanium, ensuring microscopic precision that reduces the risk of infection [17,18]. Moreover, the use of osteosynthesis screws ensures additional implant stability, facilitating immediate loading protocols [19].

While recent studies have confirmed the technical feasibility of additively manufactured subperiosteal implants, there is still a lack of consensus regarding their clinical positioning compared to zygomatic implants or traditional bone augmentation [20,21]. Specifically, the choice between zygomatic solutions—which carry risks of chronic sinusitis or orbital complications—and the less invasive subperiosteal approach remains a subject of clinical debate, with no established decision framework for the “grey zone” of extreme atrophy [22]. Furthermore, most existing literature consists of heterogeneous case reports, leaving unresolved questions regarding standardized complication management and long-term predictability [23].

These are designed through digital planning and are positioned and fixed externally, beneath the periosteum, using osteosynthesis screws. These dental implants feature a distinctive morphology and are available in various types (often referred to as mesh). Due to their design, which can be fully customized to meet the specific anatomical needs of the patient, they have become an effective and reliable solution.

The aim of this study is to bridge these gaps by evaluating the precision and advantages of the new generation of subperiosteal implants from both anatomical and prosthetic perspectives. By synthesizing recent evidence and presenting a representative clinical case, the authors intend to provide a clearer rationale for choosing juxta-osseous solutions and outline initial recommendations for their successful use in contemporary treatment settings. Furthermore, this work aims to provide a comprehensive synthesis of successful clinical applications and propose a preliminary decision-making protocol for a technology that is currently in a phase of clinical consolidation.

## 2. Materials and Methods

### 2.1. Systematic Review

This systematic review was conducted in accordance with the Preferred Reporting Items for Systematic Reviews and Meta-Analyses (PRISMA) guidelines for meta-analyses [24].

The protocol for this review was prospectively registered in the PROSPERO (International Prospective Register of Systematic Reviews) database under registration number CRD420251250186. Available from https://www.crd.york.ac.uk/PROSPERO/view/CRD420251250186 (accessed on 3 January 2026).

This systematic review of the literature aims to highlight the clinical validity of using iuxta-osseous implants in the rehabilitation of subjects with severe maxillary and mandibular atrophy.

### 2.2. Eligibility Criteria

The selection of studies for this review was strictly guided by the PICO (Population, Intervention, Comparison, Outcome) framework, focusing on patients with severe bone deficiencies of the maxilla or mandible treated with custom-made juxta-osseous implants. The study population encompassed a broad clinical spectrum, ranging from severe physiological or post-extractive atrophy to complex acquired defects resulting from oncological resections, significant maxillofacial trauma, and congenital anomalies such as cleft lip and palate. By including reconstructive contexts such as total maxillectomies and post-traumatic repairs, this review addresses the versatile application of patient-specific devices in managing diverse anatomical voids. The intervention of interest specifically involved the use of next-generation, digitally designed subperiosteal implants manufactured through additive manufacturing technologies, ensuring a high degree of technological homogeneity across the analyzed cases.

The exclusion of Randomized Controlled Trials (RCTs) and comparative studies was a deliberate methodological choice necessitated by the current state of the literature. At present, available RCTs in the field of jaw rehabilitation primarily compare conventional bone grafting with endosseous implants or involve obsolete, non-digital subperiosteal technologies that do not reflect contemporary bioengineering standards. Consequently, this review focuses on case reports and case series, as these designs currently represent the primary source of clinical evidence for the specific digital workflow under investigation. Only English-language studies involving human subjects and providing definitive clinical outcomes—such as implant stability, complications, and follow-up data—were considered eligible. Review articles, pre-clinical animal studies, and technical notes without primary clinical data were excluded to maintain a rigorous focus on the clinical and mechanical performance of these advanced titanium juxta-osseous systems.

### 2.3. Search Strategy

The systematic search was carried out across the PubMed, Scopus, and Web of Science databases, selecting original research studies published in English without restrictions related to study setting or geographic location. The literature search was performed utilizing the following search terms: (*“Jaw atrophy”* OR *“Atrophic maxilla”* OR *“Atrophic mandible”*) AND (*“Subperiosteal implant”* OR *“Juxta-osseous implant”* OR *“Customized framework”* OR *“Patient-specific implant”*).

### 2.4. Data Selection Process

Relevant data were independently extracted from each included article and systematically recorded in an Excel database. Two independent reviewers (G.C. and R.S.) evaluated titles and abstracts to determine initial eligibility. In cases of disagreement, a third senior reviewer (M.C.) was consulted. Studies for which consensus could not be reached were excluded. The same reviewers subsequently conducted a full-text assessment to determine final inclusion.

### 2.5. Data Extraction

The collected variables included: (a) first author and year of publication; (b) number of cases; (c) age and sex of the patients; (d) clinical characteristics, including systemic conditions and medical/dental history; (e) classification of maxillary atrophy; (f) type of treatment performed; and (g) duration and characteristics of follow-up.

### 2.6. Qualitative Assessment and Evaluation of Reporting Quality

The methodological quality of the included studies was independently assessed by two authors using the Joanna Briggs Institute (JBI) Critical Appraisal Tools [25]. Both reviewers assigned identical ratings. The JBI checklist consists of eight items designed to evaluate the methodological robustness of quantitative studies. Each item was scored as *Yes*, *No*, *Unclear*, or *Not applicable* (Table 1).

Since the included studies consist exclusively of case reports and case series, the analysis focused on the completeness and transparency of clinical reporting rather than the assessment of intrinsic bias, which remains inherently high for this level of evidence. Based on the proportion of positive responses, the overall reporting quality for each study was categorized as high (≤49% “Yes” responses), moderate (50–69%), or low (≥70%).

### 2.7. Illustrative Case

**Figure 1:**
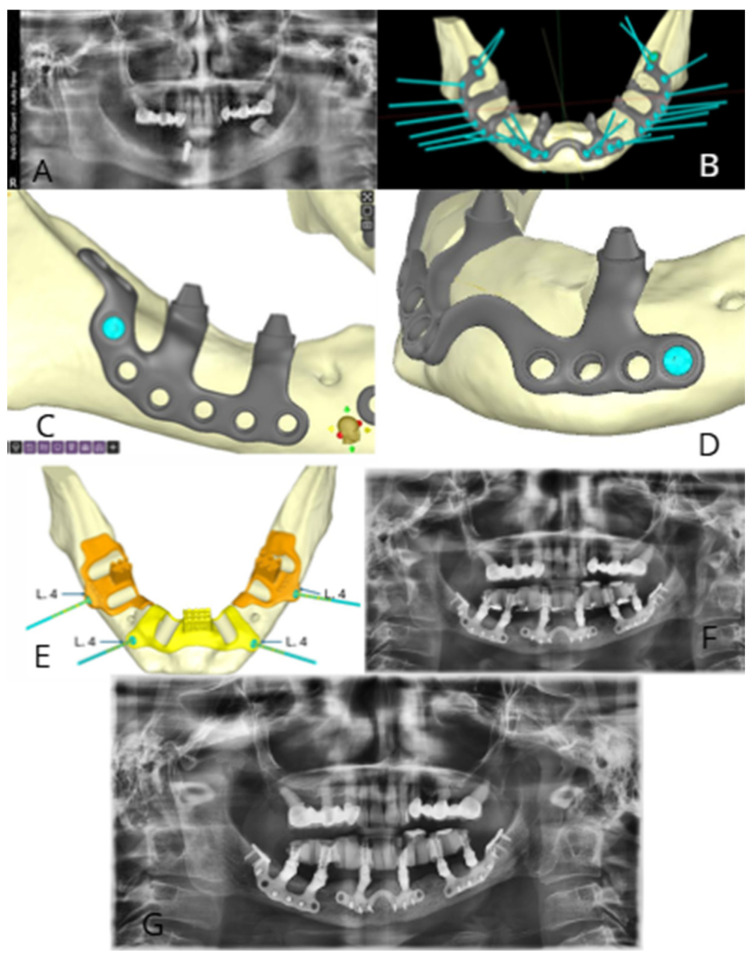
Full digital workflow and clinical outcomes for mandibular rehabilitation with a customized subperiosteal implant. (**A**) Pre-operative panoramic radiograph showing severe mandibular atrophy, a residual failing implant in region 4.3, and tooth 3.7. (**B**) Virtual 3D planning of the custom-made subperiosteal framework; the teal vectors represent the planned trajectory for osteosynthesis screws, optimized based on residual cortical bone thickness. (**C**) Detailed CAD design of the posterior implant for quadrant IV, illustrating the distal arm extension to the retromolar trigone for increased mechanical stability. (**D**) Design of the intra-mental component featuring a bridging structure across the symphysis and dedicated slots for Multi-Unit Abutments (MUAs). (**E**) Surgical cutting templates (orange and yellow) designed for the precise preparation of crestal slots to house the integrated MUAs deep into the basal bone. The teal vectors represent the insertion trajectory of the template fixation screws; the ‘L4’ notation refers to the length of the screws used for the anchoring procedure of the surgical cutting template. (**F**) Post-operative panoramic radiograph immediately following the delivery of the screw-retained provisional prosthesis, showing the passive fit of the framework. (**G**) One-year follow-up panoramic radiograph demonstrating long-term stability of the Grade V titanium implant and maintenance of the peri-implant bone levels.

The present case report is presented as an illustrative example of the digital workflow application and details the treatment of a 60-year-old female patient with no significant medical history. The patient, who utilized a removable lower denture following the failure of a prior implant-supported prosthetic rehabilitation, presented with significant functional difficulties and requested a new implant-prosthetic rehabilitation. Comprehensive clinical and radiographic evaluations were performed. Intraoral and radiographic assessment confirmed the presence of one residual implant in region 4.3 and tooth 3.7, alongside severe mandibular arch atrophy (Figure 1A).

Advanced radiographic investigations included Dental Computed Tomography (CT) scans, with section thicknesses ranging from 0.1 to 0.3 mm. A radiographic template incorporating radiopaque markers was used during the CT scanning procedure. Case planning also necessitated the acquisition of digital impressions of both dental arches and the radiographic template. The resulting DICOM and STL files were subsequently transmitted for custom device fabrication. The DICOM data was processed using dedicated guided surgery software (B&B Dental GS software https://bebdental.it/it/pro/soluzioni-digitali/software-chirurgia-guidata/ (accessed on 3 January 2026)) to accurately reconstruct the bone segments while minimizing scattering artifacts. The STL files detailing the dental arches and the prosthetic wax-up were then integrated into the dataset. The design of the custom subperiosteal implant was executed by importing the 3D files into digital modeling software (Meshmixer, Autodesk, https://meshmixer.org/ (accessed on 3 January 2026)) according to the surgeon’s specifications.

Three distinct implants were designed. Each implant incorporated supporting arms and osteosynthesis screw holes, the precise locations of which were determined based on the underlying bone thickness (Figure 1B).

Specifically, the posterior implants for quadrants III and IV featured a posterior arm extending to the retromolar trigone and an anterior arm situated near the mental nerve’s emergence from the mental foramen (Figure 1C).

Conversely, the implant designed for the intra-foraminal region comprised a structure where two arms were connected by a bridge at the mental symphysis (Figure 1D).

Crucially, all three implants were equipped with integrated multi-unit abutments (MUAs) placed in dedicated slots along the alveolar crest to position the prosthetic components deep into the basal bone. A surgical template was subsequently fabricated to facilitate the accurate preparation of these housing slots (Figure 1E).

Following design finalization, the 3D models of the bone, gingiva, prosthesis, and implants were re-imported for the surgeon’s final review and approval.

The implant, fabricated from Grade V titanium, was manufactured using Laser **Power** Bed Fusion (LPBF) technology (MYSINT100, Sisma, Piovene Rocchette, Italy). To stabilize the titanium and eliminate porosity without dimensional alteration, the implant underwent thermal treatment in a sintering oven at 840 °C for four hours, followed by an additional two hours at 500 °C. High-precision shaping of the abutments was achieved using a five-axis milling machine (Rendon GTR, Casalecchio di Reno (BO), Italy). For decontamination, the subperiosteal implant was meticulously cleaned with the organic acid DOWCLENE 1601 (Dow Chemicals Corporation, Midland, MI, USA) to remove contaminants, and subsequently sterilized.

The manufacturer also supplied milled cobalt–chromium templates for slot preparation and a stereolithographic 3D resin model of the mandible (Stratasys Objet 30, Stratasys, Eden Prairie, MN, USA) for surgical reference.

The surgical intervention was carried out under general anesthesia. Local anesthesia was additionally administered using articaine with adrenaline 1:100,000 targeting the inferior alveolar and mental nerves bilaterally. A paracrestal incision was initiated on the lingual aspect, complemented by two posterior releasing incisions at the retromolar trigone and one median releasing incision. A full-thickness flap was then elevated to achieve complete mandibular skeletonization. The mental nerve’s emergence from the foramen was identified and protected vestibullarly by careful blunt dissection of the mentalis and depressor labii inferioris muscle bundles. Lingual skeletonization extended up to the mylohyoid muscle, maintaining fiber integrity. The residual implant and tooth 3.7 were extracted prior to placing the surgical template for MUA slot preparation. After successful crestal preparation, the custom implant was trial-fitted to confirm adaptation. Definitive fixation was achieved using osteosynthesis screws of 2 mm diameter, with lengths varying from 4 to 8 mm according to the specific site.

Post-fixation, the mucosa was passively repositioned via periosteal releasing incisions and closed with 3/0 Vicryl resorbable sutures utilizing interrupted horizontal mattress stitches.

The post-operative medical regimen included antibiotic, steroid, and analgesic agents. The prescription consisted of Amoxicillin with clavulanic acid (1 g twice daily for 6 days), Betamethasone (4 mg for 2 days, tapered to 2 mg on day three), and Bromelain (1000 mg for 10 days), supplemented by pain medication as needed.

The post-operative orthopantomogram is presented (Figure 1F).

Immediate loading was performed with a fixed provisional prosthesis screwed onto the MUAs. The definitive prosthesis was delivered six months post-intervention, allowing for optimal soft tissue conditioning. The patient experienced no clinical or radiographic complications, and a 1-year follow-up orthopantomogram confirmed stability (Figure 1G). While this case demonstrated a successful 1-year stability, it serves as a clinical illustration of the protocol; these individual results should not be generalized, as outcomes in severe atrophy remain highly dependent on patient-specific factors and surgical expertise.

## 3. Results

### 3.1. Study Selection

Initially, 1918 articles were identified. After removing 467 duplicates, the remaining 1425 studies underwent manual screening based on title and abstract.

**Figure 2:**
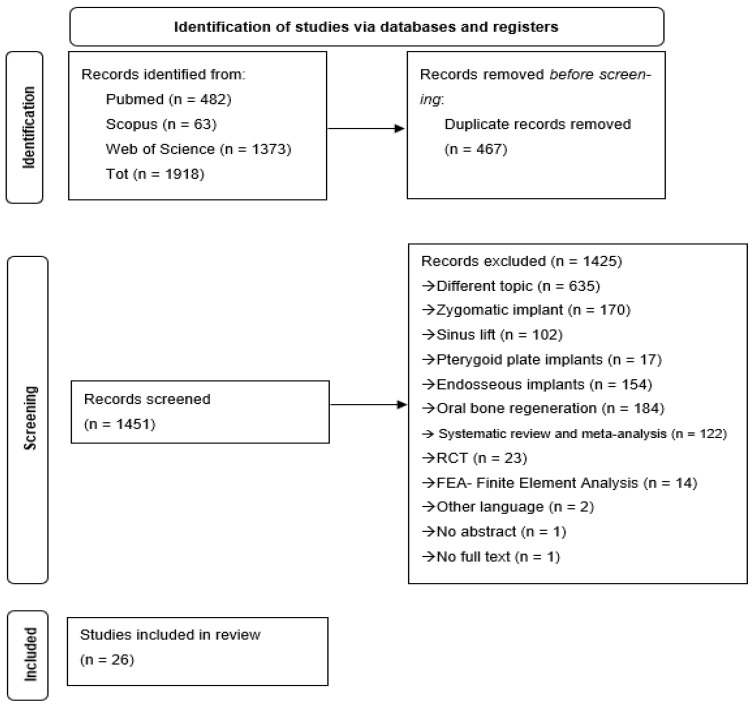
PRISMA flow diagram.

Following full-text assessment and application of the eligibility criteria, only 26 articles were included in the systematic review [7,26,27,28,29,30,31,32,33,34,35,36,37,38,39,40,41,42,43,44,45,46,47,48,49] (Figure 2).

### 3.2. Results of the Study

The 26 articles included in this systematic review and subjected to quantitative analysis accounted for a total of 147 clinical cases.

The following table (Table 2) shows the general characteristics of the included studies.

### 3.3. Age and Gender Distribution of the Patients

The demographic analysis of the sample reveals an extremely heterogeneous patient profile, reflecting the increasing versatility of personalized implant and reconstructive solutions within the modern surgical landscape. Although aggregated data confirm that the elective target remains the adult and geriatric population affected by progressive chronic atrophy, the inclusion of recent studies has significantly broadened the generational and clinical spectrum under treatment.

A female prevalence remains a consistent finding in cases of physiological or post-extractive bone resorption, as evidenced by the series reported by Lew et al., where women constituted 80% of the sample [36]. In this context, the typical candidate is a female in her sixth or seventh decade of life, with a mean age consistently ranging between 55 and 62 years.

However, the expansion of the literature to include traumatic, oncological, and malformative contexts has introduced a more balanced gender distribution and, crucially, a significant reduction in the minimum age of treated subjects. While the upper limit of the case series reaches 80 years—testifying to the sustainability of the technique even in very elderly patients—there is also evidence of effective application in pediatric patients as young as 8 years old for complex maxillofacial reconstructions. This demographic breadth is further supported by clinical cases involving young adults (aged 19–25) treated for congenital pathologies such as ectodermal dysplasia or early-onset atrophy secondary to aggressive periodontitis.

In conclusion, the subperiosteal and custom-made reconstructive approach is no longer exclusively linked to senescence; rather, it represents a versatile solution capable of addressing diverse clinical needs—from chronic bone resorption to severe post-traumatic substance loss—across a lifespan that now spans eight decades.

### 3.4. Clinical Features: Systemic Diseases and Medical History

Analysis of the clinical characteristics and medical histories within the cohort reveals a patient profile defined by severe functional impairment, frequently resulting from long-term edentulism or the failure of prior rehabilitative strategies. This is exemplified by Cardoso et al. in a case involving failed zygomatic implants [29]. Many subjects included in these studies present a history of complications related to endosseous implants that have exhausted the residual bone stock, leading clinicians such as Vatteroni et al. to opt for subperiosteal solutions in patients with insufficient volumes for conventional techniques [27].

In addition to physiological atrophy, the updated case series introduces highly complex clinical scenarios involving acquired bone defects. These include sequelae of high-energy trauma, such as gunshot wounds described Chernohorskyi et al., or oncological resections for carcinomas and ameloblastomas reported by Van Kootwijk et al., where the clinical imperative lies in restoring the structural integrity of the maxilla or mandible [41,46,47].

Regarding systemic health, while most authors select systemically healthy candidates to ensure optimal healing, a pragmatic management of risk factors, such as smoking, has emerged (e.g., Cardoso et al.) [26,29,32]. Furthermore, the literature highlights the efficacy of this treatment in patients with rare congenital conditions, such as the ectodermal dysplasia analyzed by Ayhan et al. or the hemifacial microsomia discussed by Igelbrink et al., where tooth agenesis or impaired development results in extreme atrophy from a young age [40,45].

In this context, the subperiosteal approach emerges as a definitive alternative, as emphasized by Arshad and Zwerger et al., for patients who either refuse or present contraindications to invasive autologous bone grafting [7,35]. This approach offers immediate functional stability even in cases of documented prior “reconstructive failure,” as noted by Hatamleh et al. [44].

### 3.5. Atrophy Classification

The analysis of various bone atrophy classification modalities within the examined cohort reveals a marked heterogeneity in diagnostic criteria, ranging from the application of validated scales to specific anatomical-functional descriptions for complex cases. The most frequent reference system for maxillary and mandibular atrophy remains the Cawood and Howell classification, by Diss et al. and Le Chêne et al. for Class VI cases, and by El-Sawy et al. to describe Class IV and V ridges [30,32,33].

In many studies, however, atrophy is defined not through numerical scales but in relation to the technical impossibility of proceeding with conventional endosseous implantology. This is reported by Mounir et al. and Santiago et al., who describe severely resorbed alveolar ridges unsuitable for standard implants, or by Zwerger et al., who emphasize volumetric insufficiency in both height and width [26,27,36].

A distinct category is represented by acquired or congenital bone defects that fall outside traditional physiological atrophy classifications: De Riu et al. described segmental defects resulting from maxillectomy or oncological tumor resection, while Chernohorskyi et al. classify lesions as “reconstructive failures” or total post-traumatic defects following gunshot wounds [40,41,47]. Further specificities are found in extra-dental or syndromic contexts, such as the Paprosky Type IIIb acetabular defect described by Costin et al., the irregular post-traumatic orbital volume reported by Salmi et al., or the congenital mandibular hypoplasia (Pruzansky IIb) documented by Igelbrink et al. [34,38,46].

In summary, the adoption of custom-made technology appears transversal to diverse types of bone deficits. It is applied both in contexts of classified extreme atrophy (Cawood and Howell V–VI) and in situations of massive bone loss where traditional scales prove inapplicable due to the profound destruction of the original skeletal architecture.

### 3.6. Treatment

The analysis of documented therapeutic procedures highlights a definitive transition toward integrated digital workflows, where virtual surgical planning (VSP) and additive manufacturing (AM) represent the gold standard for managing complex clinical cases. The elective protocol involves the use of customized subperiosteal implants fabricated from medical-grade titanium alloy (Ti6Al4V), typically via Laser Powder Bed Fusion (LPBF) or Electron Beam Melting (EBM) technologies, as described by Vatteroni et al., and Mounir et al. [25,26].

A hallmark of the modern technique is the capacity for single-stage surgical intervention, frequently allowing for immediate functional loading with fixed provisional prostheses within hours or days post-surgery—an approach systematically adopted by El-Sawy et al., Diss et al., and Santiago et al. [28,29,33]. Beyond titanium, the exploration of alternative materials is evidenced by the employment of polyetheretherketone (PEEK) for its superior biocompatibility and bone-like elastic modulus, as well as the use of bioactive ceramics investigated by Systermans et al. to enhance osteoconduction [28,30,50].

In advanced reconstructive scenarios, treatment evolves into sophisticated hybrid approaches: De Riu et al. describe the combination of fibula free flaps with 3D-printed subperiosteal implants, while Ayhan et al. and Hatamleh et al. integrate orthognathic surgery (e.g., Le Fort I osteotomies) with personalized reconstruction to correct severe congenital skeletal discrepancies [39,40,45]. The precision of passive fit, ensured by CAD/CAM design derived from high-resolution CT data, circumvents the need for invasive bone harvesting procedures. Furthermore, specific surgical refinements—such as the use of the Bichat fat pad mentioned by Le Chêne et al. or connective tissue grafts proposed by Olate et al.—are utilized to optimize soft tissue management and mitigate the risk of dehiscence [32,49].

In summary, the treatment constitutes a technological ecosystem merging materials engineering with precision surgery, offering bespoke solutions ranging from total dental rehabilitation to the reconstruction of extensive facial skeletal segments, such as the total mandibular replacement described by Chernohorskyi et al. [48].

### 3.7. Follow-Up

The analysis of clinical outcomes and post-operative observation periods confirms the high reliability and long-term viability of personalized solutions, with follow-up durations ranging from several months to over a decade. Longitudinal studies, such as that by Zwerger et al.—which reported a mean follow-up of 7.2 years and peaks up to 14 years—documented success rates of 75%. Similarly, Lew et al. reported stability and improved prosthetic retention in 93% of cases monitored for up to 24 months [36,37].

In more recent literature, clinical success rates have reached exceptionally high percentages: Ayhan et al. recorded a 90.4% success rate over an 18.2-month period [41]. Implant stability and the absence of infection or radiographic bone resorption remain consistent findings, as confirmed by Mounir et al. and Vatteroni et al., who highlight that the precision of the digital fit significantly contributes to optimal primary healing [26,27].

Even in complex cases of total or post-traumatic reconstruction, outcomes remain excellent: Chernohorskyi et al. reported total stability at 3 years for a complete mandibular replacement, while Fumeaux et al. and Rico et al. emphasized significant improvements in quality of life, phonetic function, and masticatory efficiency at 12 months [41,42,48]. Although minor complications have been noted—such as soft tissue dehiscence or partial framework exposure reported by Arshad, Cardoso et al., and El-Sawy et al.—these were generally managed successfully through monitoring or minor corrective interventions without compromising device stability [7,29,33].

In conclusion, follow-up data demonstrate that modern CAD/CAM technologies and additive manufacturing have transformed the subperiosteal approach into a predictable and safe procedure, capable of offering durable aesthetic and functional restoration even in the most extreme clinical challenges.

### 3.8. Evaluation of Reporting Quality

The assessment of the methodological quality of the bibliographic corpus, conducted across a total of 26 studies, reveals a profile of exceptionally high descriptive rigor, with all examined reports demonstrating superior quality scores (Table 3). This formal robustness stems from a near-systematic adherence to fundamental reporting criteria, ensuring an exhaustive description of demographic variables, baseline clinical conditions, diagnostic protocols, and implemented surgical procedures. The scientific relevance of the entire analysis is further corroborated by the meticulous documentation, present in every single study, regarding post-operative outcomes and the formulation of clinical “takeaway lessons” derived from practical experience.

Despite this general solidity, critical evaluation identified certain areas of marginal weakness, primarily related to the chronological reconstruction of patient clinical histories. While the majority of recent works fully satisfy this criterion, a lack of temporal detail persists in the contributions of Vatteroni et al. [27]. Furthermore, documentary uncertainties or the absence of explicit timelines were noted in the works of Mounir et al., Zwerger et al., Lew et al., Costin et al., and Al-Ahmari et al. [27,36,37,38,43]. An additional divergence is observed in the reporting of adverse events, which was omitted in the studies by Santiago et al., Diss et al., and Khachatryan [28,30,31]. However, it is reasonable to hypothesize that such silence reflects an actual absence of complications within successful clinical series, rather than a deficiency in the monitoring protocol. Ultimately, while surgical precision and the transparency of results confer a high degree of scientific evidence to this review, it is fundamental to recognize that the nature of the synthesized evidence remains exploratory, inherently affected by the selection and publication biases typical of case reports and clinical series.

## 4. Discussion

### 4.1. Technical Considerations and Digital Workflow

The reintroduction of juxtaosseous (subperiosteal) implants into modern clinical practice represents a fundamental paradigm shift, facilitated by the comprehensive digitalization of the surgical workflow and the development of advanced biocompatible materials [13,14,50,51,52]. The transition from analog to digital protocols has eliminated the historical limitations associated with the traditional method, which necessitated a mandatory two-stage surgical process. This former approach required an initial phase of extensive bone exposure to obtain a direct alginate impression—a material highly susceptible to dimensional distortions in a hemorrhagic surgical field—followed by a second phase for device insertion, which was frequently hindered by the need for complex intraoperative adjustments [8,12,53]. This evolution has replaced the inherent inaccuracies of manual techniques with a novel clinical perspective for treating edentulism characterized by severe bone resorption [14].

Currently, design processes based on high-precision three-dimensional radiological data (CBCT or CT) enable the fabrication of customized frameworks via CAD/CAM technologies, ensuring a millimetric passive fit against the residual bone surface [16,20]. The utilization of additive manufacturing (AM) techniques, such as Selective Laser Melting (SLM) or Direct Metal Laser Sintering (DMLS), allows for the use of Grade V (or Grade 23) titanium alloy to produce structures with microscopic precision. This drastically mitigates the risks of mechanical instability and bacterial infiltration compared to legacy cobalt–chromium alloys [6,17,21,35].

Furthermore, the integration of specialized software facilitates prosthetically driven planning, ensuring that the final implant design optimally supports the desired rehabilitative outcome from the project’s inception [15,19,48]. A pivotal aspect of this modern approach is the marked increase in patient satisfaction, stemming from the individualization of the support base and the avoidance of invasive bone grafting procedures. This simplification renders the therapeutic pathway significantly more accessible for frail patients or those presenting with systemic comorbidities [54].

Primary stability, enhanced by the strategic placement of osteosynthesis screw holes at biomechanically optimized sites, facilitates the adoption of immediate loading protocols, thereby reducing the interval between diagnosis and the delivery of the provisional prosthesis [27,50]. This streamlined workflow allows the entire protocol to be executed in a single surgical session, significantly improving subjective patient comfort and the perception of a more rapid and efficient treatment compared to traditional modalities [27,30].

### 4.2. Clinical Implications and Patient Outcomes

The analysis of updated literature facilitates a critical reconsideration of post-operative complication incidence, which historically represented the primary barrier to the widespread adoption of subperiosteal implants. The most frequently reported complication remains soft tissue dehiscence, resulting in partial exposure of the metallic framework. Recent studies, such as those by Cardoso et al. and El-Sawy et al., document cases of exposure successfully managed through minor surgical revisions or localized sliding flaps, confirming that such events, while present, do not necessarily lead to catastrophic implant failure [28,32]. Arshad advocates for vigilant monitoring in instances of micro-dehiscence, advising against aggressive interventions that could jeopardize peri-implant integration [7].

A significant trend emerging from current research is the transition toward hybrid solutions and innovative biomaterials to mitigate risks. The application of PEEK (Polyetheretherketone), as documented by El-Sawy et al. and Mounir et al., is proposed for its exceptional biocompatibility and potentially lower susceptibility to exposure compared to titanium, although the latter remains the gold standard for its superior mechanical properties [25,32]. Of particular clinical interest is the adoption of strategies to increase soft tissue thickness: Le Chêne et al. describe the use of the Bichat fat pad to isolate the implant, while Olate et al. propose palatalized incisions and connective tissue grafts to “armor” the framework, thereby drastically reducing unwanted exposures [31,47].

Furthermore, the application of this technique in post-oncological reconstruction post-traumatic scenarios demonstrates that the precision of the digital workflow allows for the successful management of profoundly altered and scarred anatomical sites [39,41,46]. In these contexts, complications are often correlated with the complexity of the primary defect rather than the device itself. The long-term stability reported in recent studies, with success rates exceeding 90%, suggests that computer-aided biomechanical load management and millimetric passive fit have effectively addressed the perimplant bone resorption issues characteristic of the “legacy era” of juxtaosseous implants [39,42].

### 4.3. Limitations of the Study

Despite the significant technological advancements and documented clinical success of modern juxta-osseous systems, this systematic review is characterized by specific limitations that necessitate a cautious interpretation of the findings. A primary constraint is the reliance on exploratory, low-level evidence, as the current study design exclusively synthesizes case reports and case series. This methodological orientation was dictated by the contemporary landscape of the literature; since the reintroduction of juxta-osseous implants is strictly linked to recent digital innovations such as CAD/CAM design and SLM/DMLS manufacturing, randomized controlled trials (RCTs) specifically evaluating these modern workflows have yet to be established. Consequently, the strength of the conclusions regarding long-term clinical predictability remains limited by the absence of high-level comparative data.

Beyond the evidence level, several intrinsic limitations of the procedure itself must be considered, starting with the steep learning curve required for clinicians, who must possess advanced proficiency in both maxillofacial anatomy and digital design software to ensure accurate planning. Furthermore, the precision of the customized framework is entirely dependent on the quality of the radiological data, as high-resolution CBCT scans free from metallic artifacts are indispensable for reliable modeling. From a clinical perspective, the extensive periosteal reflection required for implant seating often results in significant postoperative morbidity, frequently necessitating the use of corticosteroids to manage edema and improve patient comfort. Additionally, the high degree of customization and the integration of additive manufacturing technologies entail overall surgical costs that may exceed those of conventional implant therapies. Moving forward, the long-term viability of these devices will depend on a rigorous multidisciplinary approach that balances surgical precision with meticulous prosthetic design and soft-tissue maintenance. Future research should therefore transition from retrospective observations toward large-scale prospective trials with standardized protocols to definitively validate the role of this promising alternative in the management of severe bone atrophy.

## 5. Conclusions

The present review and clinical case confirm that juxta-osseous implants have evolved from an unpredictable, technically demanding solution into a reliable treatment option for patients with severe maxillary or mandibular atrophy. This evolution is primarily driven by the transition from analog, manually adapted frameworks to fully digital, patient-specific implants designed using high-resolution imaging and CAD/CAM technology.

Compared with early-generation subperiosteal implants, modern devices appear to offer a feasible alternative for managing severe maxillary or mandibular atrophy, potentially reducing treatment times by avoiding complex bone grafting.

However, it is imperative to emphasize that the present synthesis focuses exclusively on exploratory, low-level evidence, as the recent technological reintroduction of this digital workflow (CAD/CAM and SLM/DMLS) currently lacks high-level randomized controlled trials (RCTs). Consequently, the findings presented must be interpreted with due caution. Specifically, the long-term biological behavior, mechanical durability, and comparative effectiveness of these systems remain currently unknown. While long-term prospective trials are essential to establish definitive clinical predictability and standardized protocols, modern juxta-osseous implants currently represent a valid and effective alternative to conventional reconstructive implant therapies in cases of severe bone atrophy.

## Figures and Tables

**Table 1:** Checklist of Quality System Tools.

Checklist
1. Were the Patient’s Demographic Characteristics Clearly Described? Yes/No/Unclear/Not Applicable
2. Was the Patient’s History Clearly Described and Presented as a Timeline? Yes/No/Unclear/Not Applicable
3. Was the Current Clinical Condition of the Patient on Presentation Clearly Described? Yes/No/Unclear/Not Applicable
4. Were Diagnostic Tests or Assessment Methods and the Results Clearly Described? Yes/No/Unclear/Not Applicable
5. Was the Intervention(s) or Treatment Procedure(s) Clearly Described? Yes/No/Unclear/Not Applicable
6. Was the Post-Intervention Clinical Condition Clearly Described? Yes/No/Unclear/Not Applicable
7. Were Adverse Events (Harms) or Unanticipated Events Identified and Described? Yes/No/Unclear/Not Applicable
8. Does the Case Report Provide Takeaway Lessons? Yes/No/Unclear/Not Applicable

**Table 2:** Summary of Clinical Studies on Custom-Made Subperiosteal Implants.

ID	Author/Year	Cases	Patients	Medical History	Atrophy Class.	Treatment	Follow-Up
1	Arshad/2023 [7]	1	25 y male	Aggressive periodontitis; failed full-arch prosthesis after 5 years.	Severe alveolar atrophy; extraoral bone grafting declined.	3D-printed subperiosteal implant (Ti6AL4V-ELI); single stage.	3 years: satisfied with function/aesthetics.
2	Mounir 2018 [25]	10	Adult	Severe atrophic anterior maxillary ridges; no systemic diseases.	Severe anterior maxillary atrophy.	Virtual planning; CAD/CAM titanium and PEEK implants.	12 months: stability and patient satisfaction.
3	Vatteroni 2025 [26]	18	61.9 ± 6.7 y	Severe maxillary/mandibular atrophy; systemic diseases excluded.	Severe jaw atrophy.	Custom 3D-printed titanium implants; SIO success criteria.	2 years: implant survival and success assessed.
4	Santiago 2025 [27]	3	68 f, 50 m	Dissatisfied with removable prostheses; impaired masticatory function.	Severe maxillary atrophy.	Sintered titanium implants; immediate provisional rehabilitation.	3–12 months: documented prosthetic function.
5	Cardoso 2025 [28]	1	62 f (smoker)	History of failed zygomatic implants; chronic inflammation.	Severe maxillary atrophy.	Removal of zygomatic implants; custom titanium subperiosteal implant.	18 months: stable after managing partial exposure.
6	Diss 2025 [29]	1	71 f	Completely edentulous; seeking fixed rehabilitation.	Cawood/Howell Class VI.	Two implants in Ti-6Al-4V via metal laser sintering.	Postoperative stability; no complications.
7	Khachatryan 2023 [30]	1	52 f	Atrophic maxilla with significant edentulism.	Significantly atrophic edentulous maxilla.	Selective Laser Melting titanium implant; passive fit.	1 year: restoration remained functional.
8	Le Chêne 2025 [31]	1	74 f	Peri-implantitis on 6 maxillary/5 mandibular implants.	Cawood Class VI.	Customized subperiosteal implants; Bichat fat pad for isolation	1 year: no dehiscence or loosening detected.
9	El-Sawy 2024 [32]	4	65–75 y	Completely edentulous; no systemic diseases.	Cawood/Howell IV and V.	PEEK implants via CAD-CAM; 10 screws fixation.	12–24 months: functionally stable.
10	Salmi 2012 [33]	1	67 m	Orbital volume displacement after severe accident.	Increased orbital volume with ragged wall.	DMLS titanium orbital reconstruction implant.	3 weeks: eyeball displacement diminished.
11	Kusek 2009 [34]	1	80 f	Mandibular denture instability; non-contributory history.	Severe mandibular atrophy.	SLA model used for digital workflow; laser incision.	1 year: successful stability and soft tissue.
12	Zwerger 2007 [35]	12	Mean 62 y	Chronic edentulism; failed conventional rehabilitation.	Extreme jaw atrophy.	Traditional laboratory casting or CT-derived models.	Mean 7.2 years: 75% cumulative success rate.
13	Lew D. 1991 [36]	15	43–71 y	Long-term edentulism; severe ridge resorption.	Severe atrophic alveolar ridges.	Hydroxyapatite-coated implants with filling ports.	6–24 months: improved prosthetic retention.
14	Costin 2013 [37]	1	59 f	Failed hip arthroplasties; acetabular bone defect.	Paprosky Type IIIb defect.	Custom acetabular cage in Ti6Al4V.	24 months: improved mobility/quality of life.
15	Ayhan 2024 [38]	1	20 m	Hypohidrotic ectodermal dysplasia; anodontia.	Severe atrophy; Class III skeletal relationship.	Le Fort I advancement + custom titanium implant.	1 year: stable skeletal correction.
16	De Riu 2025 [39]	1	Adult	Malignant tumor requiring total maxillectomy.	Total maxillectomy defect.	Fibula free flap + additively manufactured implant.	Successful reconstruction; improved recovery.
17	Rico 2022 [40]	1	48 f	Congenital cleft lip/palate; multiple failed grafts.	Severe atrophy from cleft palate sequelae.	100% digital workflow; custom titanium implant.	1 year: excellent stability and phonetics.
18	Ow 2016 [41]	1	39 m	High-energy trauma (gunshot wound).	Severe acquired bone defect (mandible).	Custom PSI in Ti6Al4V; osteosynthesis screws.	12 months: improved masticatory function.
19	Al-Ahmari 2015 [42]	1	22 m	Large Ameloblastoma requiring mandibular resection.	Segmental mandibular defect.	EBM technology; CT data processing for fit.	Accurate fit restored facial symmetry.
20	Ayhan 2024 [43]	17	Mean 55.4 y	Severe atrophy where bone grafting was not feasible.	Severe maxillary and mandibular atrophy.	3D-printed titanium implants via CAD/CAM.	Mean 18.2 months: 90.4% success rate.
21	Hatamleh 2016 [44]	1	25 f	Severe facial deformity after ameloblastoma resection.	Segmental mandibular defect.	Single-stage orthognathic surgery + SLS implant.	12 months: stable skeletal correction.
22	Igelbrink 2020 [45]	1	19 m	Hemifacial microsomia (Pruzansky IIb).	Congenital mandibular hypoplasia.	PSI manufactured from medical-grade titanium.	Restoration of facial symmetry achieved.
23	Van Kootwijk 2022 [46]	1	50 m	History of carcinoma; failed fibula free flap.	Segmental mandibular defect.	”Digital Twin” approach with FEA analysis.	Technical validation of biomechanical reliability.
24	Chernohorskyi 2021 [47]	1	43 m	Total loss of function after gunshot wound.	Total mandibular defect.	Total titanium PSI via virtual 3D planning.	3 years: excellent stability and swallowing.
25	Olate 2025 [48]	2	64 f, 68 f	Failed conventional prosthetic rehabilitation.	Severe maxillary atrophy.	Palatal-shifted incision to avoid exposure.	6 months: healthy periimplant mucosa.
26	Systermans 2024 [49]	13	8–78 y	Oncological resections or congenital anomalies.	Complex maxillofacial bone defects.	Bioactive ceramic HA implants; scaffold-based.	Median 12 months: successful integration.

**Table 3:** Evaluation of Reporting Quality of the included studies the Joanna Briggs institute critical appraisal checklist for case reports.

St. No	First Authors Name/Year of Publication [Reference Number]	1. Were the Patient’s Demographic Characteristics Clearly Described	2. Was the Patient’s History Clearly Described and Presented as a Timeline?	3. Was the Current Clinical Condition of the Patient on Presentation Clearly Described?	4. Were Diagnostic Tests or Assessment Methods and the Results Clearly Described	5. Was the Intervention(s) or Treatment Procedure(s) Clearly Described?	6. Was the PostIntervention Clinical Condition Clearly Described?	7. Were Adverse Events (Harms) or Unanticipated Events Identified and Described?	8. Does the Case Report Provide Takeaway Lessons?	The Overall Risk of Bias
1	Arshad/2023 [7]	Yes	Yes	Yes	Yes	Yes	Yes	Yes	Yes	**L**
2	Mounir 2018 [25]	Yes	Some concern	Yes	Yes	Yes	Yes	Yes	Yes	**L**
3	Vatteroni 2025 [26]	Yes	No	Yes	Yes	Yes	Yes	No	Yes	**L**
4	Santiago 2025 [27]	Yes	Yes	Yes	Yes	Yes	Yes	Yes	Yes	**L**
5	Cardoso 2025 [28]	Yes	Yes	Yes	Yes	Yes	Yes	Yes	Yes	**L**
6	Diss 2025 [29]	Yes	Yes	Yes	Yes	Yes	Yes	No	Yes	**L**
7	Khachatryan 2023 [30]	Yes	Yes	Yes	Yes	Yes	Yes	No	Yes	**L**
8	Le Chêne 2025 [31]	Yes	Yes	Yes	Yes	Yes	Yes	Yes	Yes	**L**
9	El-Sawy 2024 [32]	Yes	Yes	Yes	Yes	Yes	Yes	Yes	Yes	**L**
10	Salmi 2012 [33]	Yes	Yes	Yes	Yes	Yes	Yes	Yes	Yes	**L**
11	Kusek 2009 [34]	Yes	Yes	Yes	Yes	Yes	Yes	Yes	Yes	**L**
12	Zwerger 2007 [35]	Yes	Unclear	Yes	Yes	Yes	Yes	Yes	Yes	**L**
13	Lew D. 1991 [36]	Yes	Unclear	Yes	Yes	Yes	Yes	Yes	Yes	**L**
14	Costin 2013 [37]	Yes	Yes	Yes	Yes	Yes	Yes	Yes	Yes	**L**
15	Ayhan 2024 [38]	Yes	Yes	Yes	Yes	Yes	Yes	Yes	Yes	**L**
16	De Riu 2025 [39]	Yes	Yes	Yes	Yes	Yes	Yes	Yes	Yes	**L**
17	Rico 2022 [40]	Yes	Yes	Yes	Yes	Yes	Yes	Yes	Yes	**L**
18	Ow 2016 [41]	Yes	Yes	Yes	Yes	Yes	Yes	Yes	Yes	**L**
19	Al-Ahmari 2015 [42]	Yes	Yes	Yes	Yes	Yes	Yes	Yes	Yes	**L**
20	Ayhan 2024 [43]	Yes	Yes	Yes	Yes	Yes	Yes	Yes	Yes	**L**
21	Hatamleh 2016 [44]	Yes	Yes	Yes	Yes	Yes	Yes	Yes	Yes	**L**
22	Igelbrink 2020 [45]	Yes	Yes	Yes	Yes	Yes	Yes	Yes	Yes	**L**
23	Van Kootwijk 2022 [46]	Yes	Yes	Yes	Yes	Yes	Yes	Yes	Yes	**L**
24	Chernohorskyi 2021 [47]	Yes	Yes	Yes	Yes	Yes	Yes	Yes	Yes	**L**
25	Olate 2025 [48]	Yes	Yes	Yes	Yes	Yes	Yes	Yes	Yes	**L**
26	Systermans 2024 [49]	Yes	Yes	Yes	Yes	Yes	Yes	Yes	Yes	**L**

## Data Availability

The data presented in this study are available on request from the corresponding author due to privacy.
